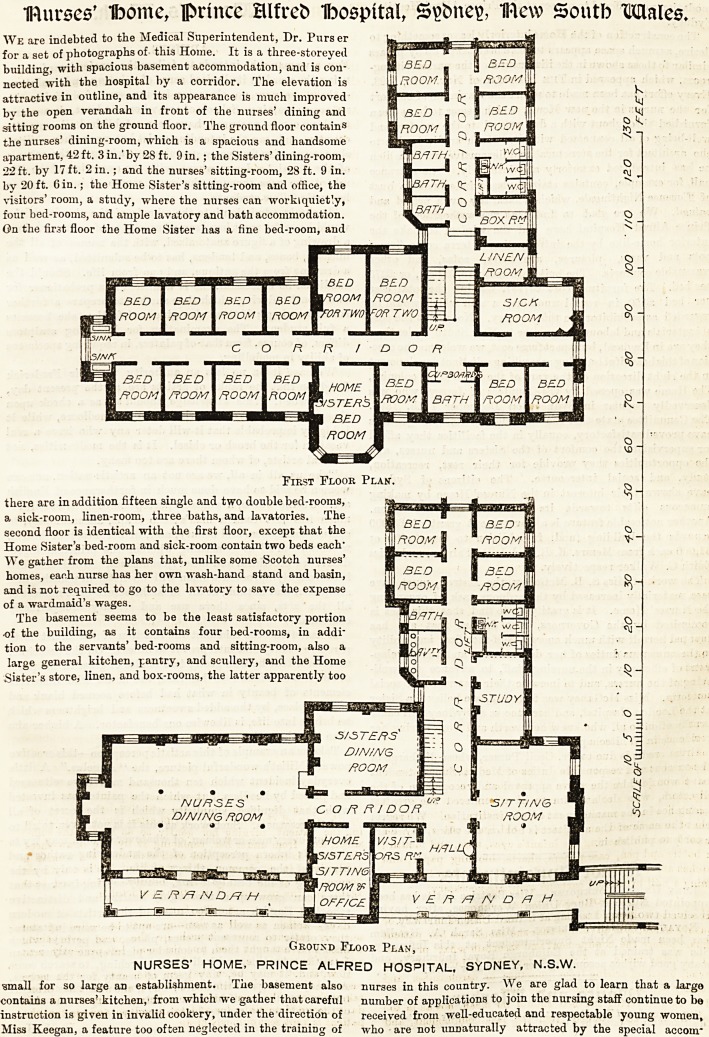# Extra Supplement.—The Nursing Mirror

**Published:** 1894-02-17

**Authors:** 


"The Hospital" Nursing Mirror.

Being the Extra Nursing Supplement of "The Hospital" Newspaper.

[Contributions for this Supplement should be addressed to the Editor, The Hospital, 428, Strand, London, W.C., and should have the word "Nursing" plainly written in left-hand top corner of the envelope.]

### News from the Nursing World.

### PRINCESS BEATRICE'S DAUGHTER.

Universal regret has been felt on account of the accident to Princess Ena, the only daughter of Princess Beatrice and Prince Henry of Battenberg. The little Princess was six years old last October, and is a general favourite. She was thrown from her pony in the grounds of Osborne House on Saturday, and was unconscious during most of Sunday. Dr. Powell, one of the Queen's physicians, is in attendance, and the little Princess is being nursed by a member of the London Hospital nursing staff. The Queen has postponed her departure from Osborne on account of the Princess's accident. Perfect quiet is still necessary, although the little patient is making satisfactory progress.

### ADDITIONS AND IMPROVEMENTS.

A capital arrangement found its place in the designs of the Great Northern Central Hospital in Holloway Road, in the shape of small pantries attached to each ward as well as kitchens and bath-rooms. These pantries or larders are lined throughout with glazed tiles, and have marble slabs for the butter, milk, &c., of the patients. There are also capital cupboards, well lighted and ventilated, for receiving the patients' clothes. The isolation wards are admirably planned, and are some of the most attractive in position and brightness. They are absolutely separate from the rest of the building, and have a special promenade retained for convalescents on a flat roof, from which a wonderfully extensive prospect is secured. It is to be hoped that the projected bazaar and plenty of liberal subscriptions will enable this most useful hospital to open its new wards quite free of debt.

### UNCOMMONLY HOPEFUL!

The simple trustfulness of some Boards of Guardians furnish those who read their advertisements for nurses with unfailing admiration. At a Welsh infirmary, for instance, a trained gentlewoman is invited to take up the duties of Lady Matron at £20 per annum. The South Molton Guardians want a nurse capable of carrying out the duties prescribed by the Local Government Board for the same salary. No applicants having appeared, they are now offering £25! Can any of these Guardians—and we have only quoted a sample of many advertisements—hope for suitable candidates? Perhaps they are really unwilling to entirely abolish the employment of imbecile and incompetent paupers hitherto the nominal but irresponsible caretakers of the unfortunate paupers who happen to be sick.

### A SUCCESSFUL SOCIETY.

An excellent report of the Leeds Trained Nurses' Institution was presented at the recent well-attended annual meeting. Of the 129 nurses now on the staff, ten are engaged in district, 102 in private work, and seventeen are still in training at hospitals. The loyalty and *esprit de corps* which are characteristic of these nurses doubtless owe their origin largely to the tact and ability of Miss Dawson, who has held the office of Lady Superintendent ever since the Home was first started. The additional Home at Wood Lane, Headingley, is found most valuable for nurses requiring change or short periods of rest. The Nursing Society possesses an Institution Trust Fund, associated with the Royal National Pension Fund for Nurses, which provides an additional £10 per annum for each nurse who has a good record of work done, when her pension becomes due.

### AN EXCELLENT RESULT.

The work of training ladies "to act as" nurses, which the St. John Ambulance Association has been carrying on at Wellingborough, has led to most excellent results, for the inhabitants of the neighbourhood are going to institute a fully-trained district nurse for the service of the sick poor. She will be supplied through the Queen's Jubilee Institute, and therefore work on the lines of the other associations affiliated therewith. We cordially congratulate the committee on their decision, and feel sure that the new nurse will find her duties facilitated, and not hindered, by all those ladies who have learnt to act as nurses in emergencies.

### A FRIEND TO NURSES.

Dr. Bilbroth, who passed away at Vienna last week, will long be remembered with gratitude by nurses, and those of his own city especially. Carrying out his ideal of efficiency, he held that nursing should be equally excellent in its way to the best medical treatment, and he therefore founded a school for nurses in Vienna, which, up to this time, had possessed no such institution. He further showed his interest in nursing matters by writing a text-book on the subject, which, through the medium of translation, is well known in English nursing circles.

### COURSES OF LECTURES.

Correspondents constantly ask for particulars as to courses of lectures on nursing which amateurs can attend. They will find particulars of these frequently given under the heading "Where to Go." There are plenty of popular courses where, after three or four lectures, the holder of the class holds examinations and awards "certificates." However, for women anxious to attend thoroughly useful and complete courses of nursing lectures, increased facilities are now to be afforded. There are lectures at St. George's and St. Mary's Hospitals, and at the Trained Nurses' Club, 12, Buckingham Street, as well as those at the London Hospital, which have always been utilised by outsiders as well as by the residents. The example of the Mile End Infirmary Committee in securing a special examiner (Mr. Percy Dean, F.R.C.S.) to judge their probationers is one which might advantageously be followed elsewhere.

### INTELLIGENT OBSERVATION.

The state of the bath-rooms is a fair criterion of the efficient or inefficient nursing in most hospitals. In one institution jams and pickles are installed in a bath-room even at the present day, whilst in another a can of milk, a cage of doves, and a pile of soiled towels formed a picture which made the bathing of patients appear a somewhat distant possibility. It would be an advantage to nurses and patients at many small hospitals if the visiting committee would make an intelligent study of ward bathrooms and kitchens as well as of the conspicuous and well-polished central tables. In fact, they should copy the surgical nurse who judges accurately of the kind of surgery in a strange hospital by the dressing baskets, splints, and appliances, because she has eyes trained to observation. On the other hand, the designer of a receptacle for milk and butter which was recently pointed out at an institution as "so cool and convenient" was evidently blind to the obvious danger of the safe being placed in close proximity to the sink—in fact, it could only be reached by the nurse stretching her arm over it.; such an arrangement showing an absence of both observation and intelligence!

### CONFLICTING CLAIMS.

"So many educated people come to the lectures," and "the room was half filled by the ladies of the neighbourhood," are common remarks nowadays. The health lecturer, trained to meet the requirements of the rural districts in which the County Council pays her to teach, constantly finds herself confronted by the difficulty of merging the "friendly chat" on nursing or health into an elaborate discourse on hygiene to meet the desires of the gentry. The latter have probably already dabbled a little in physiology, and perhaps know "the names of the bones" from a series of fashionable drawing-room addresses. They wish, therefore, to pick up a little more knowledge economically. Instead of getting a qualified teacher and paying her to instruct them systematically, they take every opportunity of attending the classes which the County Council provides for those too poor to pay the fees. This not only raises difficulties for the lecturer, but it also diverts her attention from her legitimate audience. If one of the few cottagers present inquires in a modest whisper how her Jimmie's chilblains should be treated, she is unheard because Lady S. claims the lecturer's opinion on the opposing claims of hypnotism and "Drinking the Waters at Ems." Finally, the poorer women unanimously agree in becoming silent and respectful observers, not of the lecturer's practical demonstrations, but primarily of "the quality's," fine clothes, and clever questions!

### WHERE ARE THE NURSES?

A French doctor has been writing very vigorously regarding the evils of patients' visitors in Parisian hospitals. He blames them for raising temperatures, for introducing extraneous articles of food, for noise, &c. Surely all these accusations should be levelled at the administration of the institutions in question, and not at the patients or their friends. Proper supervision in the wards, and a code of rules for all strangers who enter, would prevent most of the evils of which the doctor complains. If the visitors, after warnings, transgress by bringing in food, they should not be allowed in the wards again. But a well-organised nursing staff generally sees to the strict carrying out of the set of rules which should be conspicuously displayed in all parts of the hospital. As regards serious operation cases, of which the French doctor makes special mention, it is only necessary for the surgeon to say that his patient will be better without visitors for a few days to secure for his case the quiet isolation desired. Intelligent attention to orders on the part of the nurses seldom fails to keep the "visitor grievance" within manageable bounds. Tactful requests to the friends to co-operate with doctors and nurses in obtaining a cure are frequently acceded to cheerfully by relations, who naturally rebel against their dear ones being incarcerated indefinitely in a hospital out of their sight and knowledge.

### THE AMERICAN AMATEUR.

Under the title of "Convalescent Nurses," a class of attendants have been educated in various cities in the United States to supplement, but in to way to supplant, trained nurses. These young women receive instruction from qualified teachers which enables them, as their certificates state, only to take care of convalescents, chronic invalids, feeble elderly persons, and little children. The same instruction can be obtained by those who wish to become more useful in their home circles. Great care appears to be taken to prevent any misconception as to the differences existing between certificated nurses and those who are merely certificated attendants.

### CO-OPERATION FOR MALE NURSES.

In another column there appears this week a letter from a male nurse pathetically appealing for the benefits of co-operation to be extended to his sex. There is no reason why he and his fellow nurses should not form an association, take their own fees, and establish a general standard of training; but the last-mentioned detail, of the first importance in such a scheme, is a supreme difficulty. At present in England there is no training-school whatever for men attached to any general hospital or infirmary. In special hospitals or in asylums where men are employed they learn how to nurse patients suffering from certain diseases only. Complete instruction in the nursing of men in every kind of illness, surgical and medical, as well as mental, must be granted to English male attendants before there can arise a supply of men nurses sufficiently numerous to make co-operation practicable as well as advantageous to individuals.

### SHORT ITEMS.

Miss Ursula Baring has given £1,000 to endow a cot at the East London Hospital for Children at Shadwell.—The Marchioness of Londonderry recently presided over a successful meeting of the Newtownards Society for Nursing the Sick Poor. She congratulated the committee on their financial report.—A District Nursing Association has been recently formed at Enniscorthy, in affiliation with the Queen's Institute. —Princess Christian has promised to open the R.B.N.A. new offices in Harley Street on 24th inst.— A trained Matron and nurses are to be shortly installed at the Montrose Infirmary. The present Matron has worked for thirty-two years in the institution, and it is to be hoped that she is entitled to a pension as well as to the gift of £75 voted by the Infirmary Committee.— A very pleasant entertainment was given at the beginning of the month to the nurses at Kilmarnock Infirmary very cordial appreciation of the good work done by Miss Bowman, the matron, in promoting a system of trained nursing.

### On Nursing the Nervous and Insane.

By T. DUNCAN GREENLEES, M.B.Edin., Medical Superintendent, Grahams Town Asylum, South Africa.

### VI.—IDIOCY AND IMBECILITY.

THESE forms of mental disease, or defect, are either born with the individual or commence in very early life, before the brain has fully developed. In imbecility the growth of the brain is arrested, sometimes by convulsions in childhood, or by fits while teething, or by some of the fevers. An idiot, on the other hand, is born an idiot: the growth of the mental powers is arrested at birth, and the brain never grows again, so that in such cases education is useless.

The power of speech is one of the earliest faculties developed, and an idiot is a person who cannot speak, and who has never learned the art of language; an imbecile is a person who can speak, it may be very imperfectly, but whose mental faculties remain as in childhood, even although he may attain the bodily development of a man. His character is always childish, his habits are those of a child, and he requires the same amount of humoring and attention. He has to be treated with great forbearance and kindness, for he has no control over his feelings and emotions, and he is just like other children in being liable to outbursts of uncontrollable and causeless passion. Of course in this condition, as in other diseases, there are degrees; from the person who is simply slightly weak minded to the helpless imbecile there is a great difference; nevertheless, the condition is the same, and the question here is only one of degree.

In the other forms of mental disease the brain has attained to the limits of its development, and from some cause or other its functions have become deranged or diseased.

(1) *Mania or Mental Exaltation.*—In this disease the disinguishing characters are excitement of manner, rapidity of movement, noisy and frequently incoherent conversation; the general conduct may be fussy, extravagant, and boisterous, and the habits are frequently depraved. In these cases the controlling will-power has lost its influence over the other functions of the brain, chiefly motor, and, as a result, these functions, like an engine whose regulator is broken, play without any control.

In acute mania the symptoms are most marked. The patient is sleepless; his strength, seemingly enormous, is in reality short lived, and there is great and rapid tissue waste. This waste of tissue is seldom counterbalanced by good feeding, and the result is that the system rapidly gets run down, and an almost typhoid condition supervenes, unless the symptoms are alleviated. Cases of mania are called chronic when the symptoms have lasted for some time, and in them the excitement, &c., is less marked and violent. Asylum physicians are accustomed to look upon cases of mania as chronic where the disease has lasted for two years or upwards. There are certain patients in asylums, or for that matter outside asylums, who have delusions or ideas of an exalted or happy character. These persons are neither dull nor depressed, neither are they excited to any marked degree. They are the subjects of delusional mania, and their delusions, as a rule, influence their habits and conduct.

(2) *Melancholia or Mental Depression.*—Here the symptoms are the exact opposite of those I have been describing. Dulness and unhappiness are the chief characteristics of the minds of melancholic patients. In the acute variety they are extremely wretched; they are unable to remain at peace for any length of time; they display the greatest fear or terror, wringing their hands and moaning continuously. They dread some impending calamity, or they believe they have committed an unpardonable sin, and that their souls are doomed to everlasting punishment. They are rarely violent, but may resist stubbornly anything and everything that is done for their good. They resist being undressed at night, and again in the morning they struggle vigorously against being dressed. They refuse food, perhaps believing it to be poisoned, and it would seem in some cases that their sole aim is self-destruction, although in other cases their great fear is death, and they are constantly bemoaning the fact that some calamity is about to occur whereby they will be destroyed. As in acute mania so in this condition the system soon runs down unless sleep is procured and the mental symptoms allayed.

In the chronic form of melancholia the symptoms are similar in character, but not so intense as in the acute form. Occasionally these patients are liable to short lived attacks of cheerfulness, sometimes even passing into excitement, but they soon return to their unhappy condition. Reserved in their habits, despondent in their conversation, gloomy in all their ideas, deficient of interest in life or in their surroundings, these patients are easily recognised. They seem to be completely absorbed in their own thoughts, brooding, it may be, over some imaginary wrong they have done, or some sin they are guilty of; in some cases they are quietly maturing means to elude the vigilance of their nurses, and do them, or themselves, an injury. They sleep badly, nutrition is impaired, they lose flesh, have a sallow complexion, and they are frequently affected with constipation. They are indifferent to the quantity and quality of the food given them; and, if not carefully watched, they will starve themselves. They require rousing from their lethargic, indifferent condition, otherwise there is great tendency for chronic melancholia to slowly merge into incurable dementia.

In delusional melancholia the depression and despondency are only moderately evident as a rule; the patient, in this form, expresses delusions of a melancholic character without these delusions affecting, to any marked extent, his conduct. Thus, while they may consider themselves the most miserable creatures alive, they enjoy life fairly well, and may take an active part in the entertainments got up to amuse them. Their delusions are often of a suspicious kind, *i.e.*, that they are the objects of divine or human persecution. Sometimes they become homicidal, as, for example, when they think some one is conspiring or plotting against them.

While the suicidal tendency is one of the chief characteristics of melancholia, sometimes the natural love of life is excessively developed, and there is great fear lest they are about to be destroyed.

When melancholia alternates with mania, and is succeeded by a transient stage of apparent sanity, the condition is termed circular insanity.

### Notes and Queries.

### SPECIAL NOTICE.

The contents of the Editor's Letter-box have now reached such unwieldy proportions that it has become necessary to establish a hard and fast rule regarding Answers to Correspondents. In future, all questions requiring replies will continue to be answered in this column without any fee. If an answer is required by letter, a fee of half-a-crown must be enclosed with the note containing the enquiry. We are always pleased to help our numerous correspondents to the fullest extent, and we can trust them to sympathise in the overwhelming amount of writing which makes the new rules a necessity. Every communication must be accompanied by the writer's name and address, otherwise it will receive no attention.

### Queries.

(6) *Kandy.*—I should be glad of information about the hospital at Kandy and the nurses required.—*K. F.*

(7) *Prayers.*—Will some one recommend a book of prayers for ward use?—*Sister.*

(8) *Hospitals.*—Where can I get a list of hospitals, with number of nursing staff, names of medical officers, &c.?—*Ignoramus.*

### Answers.

(6) *Kandy* (*K. F.*).—The nursing at the Kandy Hospital has been undertaken by the East Grinstead Sisters.

(7) *Prayers* (*Sister*).—Write for catalogue of books to the Scientific Press, 428, Strand; it will give you the titles of two or three.

(8) *Hospitals* (*Ignoramus*).—You will find that and a great deal of other information in Burdett's "Hospital Annual."

### District Nursing.

### VII.—MATERNITY WORK.

### Practical Knowledge.

A practical knowledge of midwifery is a great help to a district nurse, even when she is not required to practice, for it enables her to render valuable assistance in emergencies, especially in country districts, where some time may elapse before the doctor can arrive. Not only must she understand the technical part, but she must be able to look after mother and infant under the most unfavourable conditions, and do her best to avert evil consequences which may ensue. A midwife and monthly nurse should be antiseptic in every detail—herself, her dress, her bag, her method of dealing with the patient, both during and after confinement, must be beyond suspicion.

### Washing Dresses.

Every maternity nurse, whether midwife or not, should wear dresses of washing material, preferably light in colour, and light aprons. The dress sleeves should unbutton at the wrists so that they can be turned up over the elbows; oversleeves are not desirable for midwifery work. No rings, except perfectly plain bands, should be worn. A bonnet without a veil and white washing strings is most suitable.

Whenever possible, a nurse should have a bath, including her hair, and change all her garments before attending as a midwife. The skin of her hands should be free from roughness and abrasions, especially the index and second fingers of the right hand, and the nails should be quite short and scrupulously clean.

### The Midwifery Bag.

The midwifery bag should be entirely distinct from the district bag, with separate bottles, scissors, &c., lined with leather, and over that a removable lining of holland or similar material which can be frequently washed.

The bag should contain an irrigator with glass nozzle (without a terminal hole), a Higginson syringe complete in a sponge bag, a gum elastic and a glass catheter (No. 7 or 8), a papier-maché receiver, blunt-pointed surgical scissors, clinical thermometer, medicine and minim measure glasses in a case, and a bath thermometer.

Glass pots with screw lids are needed for mercurialised or carbolised vaseline (1·2000 or 1·20), powder for dressing cord (equal parts zinc and boracic to two of starch powder), permanganate of potash crystals, iodoform powder, and boracic crystals.

A bottle of perchloride of mercury tabloids is useful for disinfecting the nurse's hands or for giving a douche if ordered by the doctor. A 2 oz. bottle of creoline is desirable, the same quantity of brandy, 1 oz. of liquid extract of ergot and of tincture of iodine, also a little of Valentine's meat juice or some other strong extract.

A box of strong safety pins, a packet of ordinary ones, a small tin box for the ligatures of the cord, which should be ready made of four to five strands of strong linen thread; a washing hold-all, containing threaded needles, cotton, tape, skein of linen thread, and squares of old linen cut ready for the cord and for washing the infant's eyes, nose, and mouth; ink bottle and pen, case papers, charts, blotting paper, and matches are all needed. A hand towel and soap box, containing a nail brush and carbolic soap, are necessary.

### Preliminary Suggestions.

If a nurse is engaged to attend a case either as midwife or monthly nurse, it is advisable that she should see her patient at home before the confinement and ascertain what preparation is being made. A few suggestions beforehand may prevent mischief arising from ignorance or carelessness, and certainly will save confusion at the time.

The tick covering the bed should be clean, not as is too often the case, still stained from the last confinement, and the quilt and blankets also washed. The woman should have a change of linen for herself and the bed, besides some old things that can be used at the time, enough clothes to keep the baby fresh and clean, and if she does not possess an oil-cloth table cover, a piece of waterproof should be obtained. A piece of round towelling or strong unbleached calico will be needed for the binder, also some towels, pillow-cases, &c.

The bed may be placed in a more convenient position, and the nurse can also ascertain the state of the sanitary arrangements. Every district maternity nurse should have a store of bed linen, baby clothes, &c., to lend in emergencies, and should know where to direct the mother to apply for a maternity bag, if needed, always encouraging her to pay for the same.

### The First Duty.

When called to a case, the first thing a nurse must do before touching her patient in any way is to disinfect her hands thoroughly, either in 1-2000 or creoline and water. Before making any examination the fingers must also be well covered with the prepared vaseline.

The next step is to secure plenty of hot and cold water in the room, a slop pail, two basins, and a jug holding about a quart; this is useful in case a douche is ordered, or if instruments are required to be warmed.

The bed is best prepared by folding back the top sheet, blankets, quilt, &c., to the other side of the bed; if two pieces of mackintosh are procurable one may be placed under the bottom sheet the other over it, with thickly-folded clean pieces of material on the top. This is placed at the lower end of the bed, so that the woman will be on her left side with her feet against the foot of the bed and her head on pillows in the middle.

The patient has clean night clothes put on, which are pinned securely over each shoulder, and a clean flannel petticoat and skirt; the women generally think any dirty ones will do for this purpose, but clean ones are essential. A draw-sheet or its substitute is rolled up ready and put conveniently at hand, with the binder and safety pins.

### The Infant.

The child's clothes are arranged by the fire, with threaded needles, powder, safety pins, and linen for cord, eyes, &c., and some boracic lotion for bathing the eyes, if approved by the doctor.

The brandy should be at hand in case it is suddenly needed, and a drain of ergot measured in the minim glass, with 1 oz. of water in the medicine put ready in case of need.

No midwife should administer ergot until after the conclusion of the third stage of labour, and no monthly nurse has the power to do so at any time. The receiver is convenient for the placenta, &c.

As soon as the child is born, its eyes, nose, and mouth should be carefully cleansed, and then it should be wrapped warmly in flannel, and not washed until half an hour after the ending of the third stage of labour, if the mother is in a satisfactory condition. It is then washed, special attention being paid to the eyes, and the mother is made comfortable in bed. This can be accomplished with hardly any exertion by drawing off the soiled skirts, and after thoroughly cleansing the patient, turning her gently so as to roll the soiled sheet, &c., from under her, replacing it by the draw-sheet, the binder is then put on, and with assistance the woman is easily lifted in the draw-sheet to the top of the bed with her head on the pillow. The top bedclothes are turned back again, and the patient left dry and comfortable.

### Soiled Linen.

Every article of soiled clothing must be rolled up and securely fastened into a bundle by the nurse, everything washed that has been used, and the room left perfectly neat and tidy before she goes.

It must not be forgotten that the after-care is even more essential than the confinement, and if a midwife has undertaken the case she must carry it out herself. She should visit daily, twice if possible, for at least five days, and see that the patient is kept scrupulously clean.

### Nurses' Home, Prince Alfred Hospital, Sydney, New South Wales.

We are indebted to the Medical Superintendent, Dr. Purser for a set of photographs of this Home. It is a three-storeyed building, with spacious basement accommodation, and is connected with the hospital by a corridor. The elevation is attractive in outline, and its appearance is much improved by the open verandah in front of the nurses' dining and sitting rooms on the ground floor. The ground floor contains the nurses' dining-room, which is a spacious and handsome apartment, 42 ft. 3 in. by 28 ft. 9 in.; the Sisters' dining-room, 22 ft. by 17 ft. 2 in.; and the nurses' sitting-room, 28 ft. 9 in. by 20 ft. 6 in.; the Home Sister's sitting-room and office, the visitors' room, a study, where the nurses can work quietly, four bed-rooms, and ample lavatory and bath accommodation. On the first floor the Home Sister has a fine bed-room, and there are in addition fifteen single and two double bed-rooms, a sick-room, linen-room, three baths, and lavatories. The second floor is identical with the first floor, except that the Home Sister's bed-room and sick-room contain two beds each. We gather from the plans that, unlike some Scotch nurses' homes, each nurse has her own wash-hand stand and basin, and is not required to go to the lavatory to save the expense of a wardmaid's wages.

The basement seems to be the least satisfactory portion of the building, as it contains four bed-rooms, in addition to the servants' bed-rooms and sitting-room, also a large general kitchen, pantry, and scullery, and the Home Sister's store, linen, and box-rooms, the latter apparently too small for so large an establishment. The basement also contains a nurses' kitchen, from which we gather that careful instruction is given in invalid cookery, under the direction of Miss Keegan, a feature too often neglected in the training of nurses in this country. We are glad to learn that a large number of applications to join the nursing staff continue to be received from well-educated and respectable young women, who are not unnaturally attracted by the special accom-

First Floor Plan.

Ground Floor Plan,

NURSES' HOME, PRINCE ALFRED HOSPITAL, SYDNEY, N.S.W.

modation now offered to nurses at the Prince Alfred Hospital, Sydney.

The construction of the Home interiorly leaves something to desire, as much space appears to have been taken up by pillars similar to those shown in the illustration of the nurses' sitting-room, which appeared in The Hospital of Nov. 18th, 1893. Every effort has been made to secure the maximum of comfort for the nurses in the new Home, which seems to have been furnished throughout with a due regard to the comfort and well-being of all connected with it. Much credit is due to the architect for the structural and decorative effects which he has introduced exteriorly and interiorly. The entrance hall, for example, contains stained glass windows and a bust of Florence Nightingale, which is naturally much prized and valued. We are glad to find that the authorities of the Prince Alfred Hospital have had the wisdom to make the interior home-like by the introduction of terra cotto flower pots and vases, pictures, armchairs, sofas, and other reasonable comforts. The buildings cost £13,767 4s., or £275 per bed. The furniture cost £1,731, making the total cost per bed £350 in round numbers, a sum which would be regarded as prohibitory in this country. No doubt the cost of materials and labour are greater in New South Wales than they are in England, but apart from cost, we welcome the erection of this beautiful building as marking a distinct development in the right direction on the part of the Australian colonies, The Home was opened by the Countess of Jersey, who was deservedly popular in Sydney, on December 13th, 1892. The Committee state that the building and its arrangements have proved satisfactory, equally in the facilities they afford for supervision, the comfort of the Sisters and nurses, and the opportunities they provide for their rest, recreation, study, and social intercourse. The citizens of Sydney have shown their interest in the Nurses' Home by making numerous gifts towards its furniture and decoration. Another noticeable feature is a Government grant of £5,000 towards the building fund, in addition to three gifts oj £1,000 each from Messrs. E. R. and J. R. Fairfax, and Miss Edith C. Walker respectively.

The work of Miss S. B. McGahey, the Matron, must have been materially increased by the arduous task of organising the Nurses' Home. It is gratifying to find that this fact is recognised by the Governors, who declare that she has devoted herself with much energy, self-sacrifice, and ability to the numerous duties of her department, and the development of efficiency in the nursing staff, to advance the well-being of the nurses, and to increase their material and social comforts. Miss McGahey was formerly, we believe, a Sister at the London Hospital, and her success at Sydney will cause gratification to all who knew her worth and work during her residence in Whitechapel.

Great credit is due to Dr. Cecil Purser, whose discharge of the onerous and responsible duties of Medical Superintendent have won for him the warm approval and recognition of the directors, who declare him to have rendered the greatest assistance in the management of the institution. We regret that the name of the architect is not on the plans, so we are unable to publish it.

### Minor Appointments.

Bury Union Workhouse.—Miss Beatrice Astles has been appointed Nurse at Bury Union Workhouse, after having received two years' training at Salford Union Infirmary.

Royal Infirmary, Bristol.—Miss Sarah A. Headlam has been made Night Superintendent at this Infirmary. She was trained at the Edinburgh Royal Infirmary, and many good wishes accompany her to her new work.

### Wants and Workers.

Can any reader of The Hospital tell Miss C. W. of any home to which admittance can be gained by a collection of a million postage stamps?

### Women's Work.

### ARTISTS.

For a woman who intends to follow the profession of art—using the word in its limited sense of painting and sculpture—there are now no obstacles in the way of obtaining the necessary training. Schools of art, where, at least, elementary teaching can be procured, have been established all over the country, and, can the intending artist but prove her ability, she may receive five years gratuitous teaching in the schools of the Royal Academy, where, with the exception of the life school, she goes through the same course of instruction as her male fellow-students.

To obtain admission into the schools of the Academy the would-be artist must have some knowledge of anatomy, since a drawing of a figure anatomized, with the names of all the muscles, bones, and tendons, has to be submitted, as well as a drawing from the antique, and one from life. Should the candidate be successful, she is admitted as a probationer for two months, during which time she has to prepare a further set of drawings; if these also give satisfaction, she becomes a full student. The examination for intending sculptors differs, of course, from that of painters, in requiring specimens of ability in modelling.

We have it on no less an authority than Sir Frederick Leighton that the ranks of artists are, at the present day, overcrowded. This knowledge should act as a check upon those whose talents do not rise above the mediocre, while it is highly improbable that it will deter any who have a real vocation for the brush or chisel. It is the mediocrities, not the real artists, of whom there are too many.

Take us all in all, we are not an artistic nation, nor can women be said to have done much in furtherance of English art. On the whole, a woman's perception of the beautiful is much less just than a man's; she is apt, unconsciously, to confuse uselessness and beauty, and, in her heart of hearts, to define "art" as something whereof the principal ingredients are prettiness and unserviceableness. Until this idea is thoroughly uprooted women will do little in painting and sculpture, and nothing at all in architecture—the noblest of all the arts, since there use and beauty are perfectly combined.

It has often been said that the function of the artist is to perceive beauty and interpret it to others. On the principle, therefore, that he who grows two blades of grass in the place of one is a benefactor to his race, the artist who discovers elements of beauty in what had before seemed blank and commonplace, by the added sweetness and brightness which he brings into life, is likewise our benefactor. A higher aim than this the artist cannot have.

Take as an example of this artistic perception—this creative power—Millet's wonderful picture, the "Angelus." A little everyday incident which ten thousand men have witnessed and passed by heedless, but which the painter has invested with that dignified simplicity which is the secret of all artistic greatness. Such work as this it is not given to all to do; but every man or woman, of whatever calling, may try to share his inborn perception of the underlying pathos and beauty of the common things of life. It is only by the spreading of the beauty-loving, beauty-seeking instinct that there can grow up a thoroughly healthy and distinctive school of English art. Too many of the artists of modern days—women as well as men—are apt to be mere imitators; they prefer to represent scenes, places, and periods which others have taught them to consider picturesque rather than by patient, humble, and individual searching, to search some new, and, it may be, tiny germ of beauty for themselves. Art may be long and life short; but if the artist has brought but one small gem of his own working to lay at the shrine of the goddess he serves, he has not lived in vain.

C. M. H.

### Old-Time Nurses.

### I.—A PLEA FOR THE MIDDLE-AGED.

IT is but a little over thirty years since the new race of nurses began to come into being. The pioneers in the science and art of nursing are still among us, and the generation of the orthodoxly-trained, rapidly as it increases, still far from equals the race of what, for want of a better word, we must term "old-time nurses." So much has been said and written about the necessity for a long course of systematic training in the nursing profession, and this necessity is now so generally admitted, that it may be permitted to look back and see what merits among its many defects the old system presented, what survivals from it remain, and to what extent the elder members of a very honourable profession are entitled to look for recognition at the hands of the public.

There is not much need to expatiate on the evils of the past. Nearly everyone's experience contains a specimen of the bad nurse who was very, very bad, incapable, obstinate, hopelessly untrustworthy; and some have the bitter memory of having witnessed blunders in the treatment of those dearest to them which frustrated the physician's best skill, and entailed causeless suffering, even death on the patient. Training has at any rate done away with this class. But it must not be forgotten that the old system also produced the good nurse, who was very, very good. Of this type is the Little Sister in Thackeray's "Philip," who, with her shrewd common sense, tenderness, humour, and zeal for her profession, is unquestionably the finest nurse in all literature.

The "old-time" nurse did not, as a rule, adopt her profession very early in life, and as often as not she drifted into it after testing her vocation in the illnesses of all her relations. Her experience was won either in the hospital wards (and here in midwifery at least some form of instruction was early obtainable) or, far better, under the eye of some special doctor whose directions constituted in many cases a pretty complete course of training. Very often, no doubt, neither of these introductions to nursing was obtainable, and the nurse taught herself by mother wit, and to a certain extent at the expense of her first patients. But the presence of mind, ready resource, and sound judgment, developed by years of varied experience, resulted in the acquiring by the good members of the class of a very high skill in nursing. These facts have until quite lately been fully recognised, but the rapidity with which the nursing profession is advancing has roused the danger of overlooking the services and neglecting the value of its older members. It was only by slow degrees that sound hospital training became available, even after its importance had been understood, and the number it was possible to train on the new lines was until but a short time back sternly limited. No blame of careless omission then can attach to those who now find themselves stranded in a profession to which they have dedicated the best years of their life and to which they are deeply attached.

Every day the difficulty of maintaining a place in the nursing world without the fullest hospital training increases. And in many points of view, and specially as regards the young women who enter the profession, this is just as it should be. But the extent to which the older nurses suffer is very little understood. Some idea of it may be gained, however, from a glance at our advertising columns, where the clause "not over forty" is becoming almost a stock formula. Under this clause, indeed, hundreds of nurses in addition, whose training is perfectly modern and satisfactory, find themselves shut out from work. Up to this point in their lives they may have found their services eagerly sought, and may have given the most entire satisfaction, but after passing the fatal barrier all is changed. With health unimpaired, with excellent testimonials, and with the experience and tact which only age can confer, they find themselves exposed to the humiliation of constant rejection, and reduced after long periods of idleness, during which the savings of years are consumed, to the extreme depth of destitution. This is no fancy picture. It is the history of many valuable nurses now struggling in our midst for mere sustenance, and falling a ready prey to so-called nursing agencies, which demand in commission as much as a third of their weekly wage.

It may be argued that the objection to middle-aged nurses is one founded on experience, and that they have been proved to be less capable, less intelligent, and more exacting as to personal comfort than younger women. In this respect, as in many others, the innocent suffer for the guilty. All those who have no true vocation for a profession do tend to become more slothful in the performance of its duties as years advance. And the nursing profession is peculiarly open to the invasion of the hopelessly inefficient middle-aged woman, who takes it up in the hope of finding something light and not "menial" to do, and who calls herself trained on the strength of having scrambled through a few months at some small hospital. Any advertisement which seems to promise light work will bring forth a host of answers from the most unsuitable persons, with ideas of nursing often resembling those of the kind-hearted lady who thought it must be "so nice to go up and down the ward all day, putting on a poultice here and a poultice there." The advertiser has perhaps neither time nor skill for the task of weeding out the suitable candidate from among such applicants. By limiting the post to younger women this class of incapables is certainly to a large extent excluded, but many practical and thoroughly efficient nurses are placed at a grave disadvantage. And it is not they alone who suffer. A great many patients will be found infinitely to prefer the ministrations of a quiet, elderly person, merely a comfortable presence, with no obtrusive personality, to those of an enthusiastic, self-reliant, slightly despotic young "lady." One cannot help sympathising with the officers in India who were irritated rather than soothed by the presence at their bedside of the ministering angels with whom they had danced a few nights before. There are many in England, too, who echo their longing for the "old-time" nurse, but who submit perforce to the chastened despotism of the younger women from the fear of falling into the hands of some blundering impostor. And, in addition, there are numerous cases which will occur to everyone where mature age so far from being a drawback is all but the highest recommendation.

We are brought face to face, therefore, with the following position: A large number of skilled workers for which there is an abiding demand are placed at a complete disadvantage by the co-existence of an unskilled and inefficient majority, with whom they are continually confounded. Would it not be possible for the minority to combine themselves into an association which should exclude all but the really efficient? * The public could hardly fail to take advantage of an organised band of nurses, no longer young but in no sense past work, able to show a long record of experience and possessed of first-rate testimonials to skill and conduct.

All honour to the zeal and devotion to their duties displayed by the younger members of the profession. But properly directed this first keen absorption in the interest of the work ripens into something higher and better. More tolerance, wider perceptions, sounder practice are the result of the daily discipline and self-surrender of a true nurse's life. Each year of this discipline adds its ripening touch to the character, and not till the physical powers are so materially weakened as to hinder the right performance of necessary duties should the nurse be made to feel that her day is over, and the hour of repose is at hand.

* Correspondence is invited from any nurses interested in this suggestion.

### The Nurses' Confessional.

" How poor are they who have not patience,
What wound did ever heal but by degrees."

Our nurse readers often write to us on subjects which are of general as well as personal interest, and we propose in future to insert such communications under this heading. We invite confessions of mistakes made in the course of a nurse's work, and we think those who have charge of the sick may help each other greatly by thus assisting their fellow workers to escape some of the blunders they may have made themselves.

### TRIVIAL TRIALS.

" Ask God to give her skill in comfort's art,
That she may consecrated be, and set apart
Unto a life of sympathy."

Each of us may do this daily and begin each morn with a determination that we will be strong, but when night comes and we glance back through the day, we find that we have failed miserably, not in the great things, but in the scores of trifling things which make up a nurse's day on duty. It may be that an unjust or unkind thing has been said by one whose good opinion is valued, or someone has come between two close friends and caused coldness, nay, perhaps alienation. Some favourite of the matron's or sister's may have represented to her something said in quite a different light from the intended one, so bringing down unmerited wrath. Such trivial things as these compose a reason for the rampant vice of grumbling in hospitals. This is the reason why a nurse fails in preserving, under all circumstances, a cheerful, contented spirit, in fighting down the inclination to complain of petty grievances, to forgive those who annoy. She says to herself, "It is no use my trying, it cannot always be my fault" Why do we fail in this, which is clearly a nurse's first duty? Do we not know that her life must be in many ways one of self-sacrifice? Is it not that self instead of being sacrificed comes to the front, and retards all our endeavours to be good and to do good? We need the constant disciplining of ourselves and seeking of God's grace to enable us to say daily, "Not to do mine own will, not for one day or one week, but to go on, content to wait, like the sower, for first the blade, then the ear, then the full corn, resting in God's promise that whilst His beloved sleep He will strengthen them, that in the end they may walk worthy in the vocation wherein they are called, and so bring forth fruit with patience.

### WOMEN'S RIGHTS.

A woman's rights, what do these words convey;
What depth of old world wisdom do they reach;
What is their real intent, oh, sister, say,
And strive in daily life their truth to teach.

Ministering is certainly woman's vocation, be her talents what they may, and the woman who would seek nursing as a vocation had better leave her fine ladyism and lackadaisical nonsense at home if she wishes to make any progress in nursing poor suffering humanity. Self must be put entirely in the background, and patience, gentleness, and untiring energy must be brought forward, for the life is full of stern realities and great responsibilities. Too often nurses as a rule do not fully realise the influence which in one way or another they exert over those they come in contact with day by day. How deep and lasting is the impression received from a true-hearted nurse—one who is living in daily hourly contact with the Master who went about doing good. It seems as if a nurse's responsibility is greatest in children's wards even than with adults, simply because the little characters are unformed. Surely the very fact of unconsciously moulding the characters of those little impressionable atoms of humanity should make a nurse doubly careful of her own life and conduct. If the heart is in sympathy with the little sufferers the hand and manner will be gentle. The nurses' influence may help to make children into true men and women, living noble, earnest lives, or may mould them in another baser form by an example of frivolous and self-seeking behaviour. As the farmer ploughs fields and sows his seed with a willing hand, in perfect trust that the gentle showers will descend, that the sunshine will penetrate the soil, and in due season the fruit of his labour will appear, so must the children's nurse also work in faith, not seeing but believing.

The girls that are wanted are good girls,
Good from the heart to the lips;
Pure as the lily is, and pure
From its heart to its roseleaf tips.

—J. L. J.

### Domineering Nurses.

By an Impartial Observer.

" And did you like your nurse?"

"Oh, yes, very much," said the lady whose recent serious illness had necessitated trained attendance; "but why do all hospital nurses make enemies in the house?"

"By their inherently domineering manner," promptly replied her friend; "it's a pity now, isn't it?"

A pity indeed! All true nurses will echo the remark, while they must in justice acknowledge that the charge is not without foundation. The same story is constantly heard even in these days, "Oh, anything rather than a trained nurse; they are so overbearing, they upset the whole house."

Of course there are two sides to this as to every question, and nurses may answer, "Many of these complaints result merely from ignorant prejudice on the part of the patients' friends, and servants. The latter object to the enforcement of necessary sick room rules, but sometimes nurses themselves are to blame, not for the matters in question, but for the manner in which they carry them out. Many good nurses quite fail to realise the enormous importance of courtesy in little things, unfortunately for the credit of the nursing profession. Women who have a high standard of their duty and responsibility, whose life-work is the care of the sick and suffering, should never forget what is due to themselves and the profession they represent as to lay themselves open to an accusation of "domineering."

To put the matter on no higher ground than the professional desire to do the best for a "case," can anything be more likely to retard recovery than the existence of domestic jars or skirmishes between nurse and servant?

Rules have to be made by the heads of private nursing institutions, and undoubtedly must be kept, but superintendents are well aware that each nurse must use her private judgment in individual cases, hard and fast regulations on minor matters being frequently both exasperating and harmful.

In the particular instance which we have referred to there was no accusation of "disobligingness" on the nurse's part, but behaviour which the submissive patient had evidently considered as much a part of a "hospital nurse" as apron or cap. She had made "enemies," and therefore a condition of armed neutrality existed between her and the authorities "below stairs," merely because things needed for the sick room were asked for in what was considered a "domineering manner." How many little harmful rubs and worries might have been spared the patient had never entered into the calculations of the trained nurse in charge of her. Until all nurses gain a larger measure of tactful courtesy their "training" will be incomplete, and their powers for good will be lessened and hindered.

Superior knowledge on practical points does not justify anyone in assuming dictatorial airs when dealing with those who have not had opportunities for acquiring similar experience, and hospital methods need certain modifications in applying to patients in their own homes. There would be an end to trivial rubs and annoyances if all nurses followed the worthy example of the real ministering women who avoid hampering the good work lying ready to their hand. The accusation of an overbearing manner could never be made against those true nurses who give their entire attention to promoting the welfare of their patients, leaving trivial personal matters to take the secondary position which alone is their just due.

☞ For The Nurses' Looking Glass, Where to Go, Everybody's Opinion, and Reading to the Sick, see page cxcix et seq.

### The Muses' Looking Glass.

### MAKING ACQUAINTANCE WITH MICROBES.

(BY ONE RECENTLY INTRODUCED TO THEM.)

MICROBES formed the subject of the last and most attractive of our popular lectures. We had no idea before they were such delightful, attractive, little creatures. Perhaps no one could realise it without attending some such lecture.

It gives a queer feeling to know that everywhere around us, in some form or other, the diminutive organisms exist. On each square foot of a certain district in London, our lecturer told us, hundreds of thousands fall to the ground every minute—the lady who sat next us gave a shiver at this, and whispered that she would never dare to walk in that neighbourhood again. If she ever were obliged to pass through it she would go in an omnibus, which would be safer. Another lady was really charmed. She had a very small microscope, of which she was absurdly proud, and on it she based a reputation of being scientific; she made up her mind at once to go to that locality and bring home a handful of germs to examine under her wonderful instrument. "Don't expect any of us to come near you while you have the horrid things in your house," said her friends in alarm.

It is not easy to remember much about that lecture, or even to be absolutely certain which are microbes and which are not! The amœbæ, about which our lecturer spoke a great deal, are very innocent little bits of jelly, and only just escape being vegetables. They are not dangerous, and, therefore, not interesting. The one that took our fancy most, and about which we feel no doubt whatever, was the dear little "Cock-eye." That is not his real name, of course; he is too grand for an English title, and is only spoken of in Latin as "Coccus." The plural being "Cocci," is simply anglicised, "Cock-eye." "Is he called so because he is one-sided?" asked a friend. The lecturer had drawn him very crookedly on the board. "Oh no, that is not the reason," another person answered, "it is because he is so lively, and winks one eye." The little joke fell flat. "He hasn't any eyes," objected the lady. This little Cock-eye is very sweet; he has at either end a little wavy feeler, called a flagellum; he does not use it, as one might think, to chastise the naughty little ones, but for whipping food into his own mouth—not that he has a mouth, so that is a mere figure of speech, but, really, what is one to say of a little creature that consists solely of a place into which to stow his food and of two arms to catch it with?

There is another fascinating wee organism called "Spiralis," formed like a corkscrew, rather an awkward-shaped one, except when he wishes to force an entrance where he is not wanted. The way in which these microbes invade without the least warning is really underhanded. They are so tiny, too, they cannot be seen through an ordinary microscope. Our lecturer had a microscope, and he put little glass slides underneath and told us to look. "There," he said, "that is a consumption bacillus." So one of the audience looked, and remarked, "I see beautifully; there are two or three long black things which keep moving. Are they alive?" "Nonsense," said the lecturer; "you are looking at your own eyelashes." So carefully putting her eyelashes out of the way she succeeded in seeing one or two tiny dark specks on a blue ground. "But these are not what you described to us; they are just wee dots, and have no flagellums," she remarked, resentfully. Dr. S. had drawn such entertaining little pictures, and was such a very nice man, that we did not like to think he had been drawing on his imagination as well as on the blackboard. "Oh," he answered, coolly, "this microscope is not nearly strong enough to show the details; it merely serves to give an idea of the exceeding smallness of the bacillus." All the same it was a disappointment we all felt.

The lecture was intended to teach us to beware of microbes, and not to entertain them under any circumstances, for there is no instance on record of their proving to be "angels unawares." Dr. S. explained to us how we might baulk them even though they had made an entrance into our houses. They like heat, he told us, but not of a violent character and when it comes to being boiled or baked they draw the line at it and die. It reminds one of the well-known proverb, "first catch your hare—or microbe—then cook him." Still this is a very simple matter, for he is in certainly the milk-jug, so milk must be boiled; also he is sure to be in the butter. That could not be easily boiled or baked, so one must either do without butter or be content to eat him!

Perhaps after all it is only in the abstract that microbes are lovable. Last year, when the influenza bacillus attacked us, we hated him individually. Now one lady says her life is a burden to her for fear of microbes; she boils and bakes, and disinfects and worries her friends and servants dreadfully. And yet, with all her precautions, she says she is more pursued by germs than anyone else. She took her children to the seaside a short time ago, and hunted for healthy lodgings. How difficult she found it to get a docile landlady to bear with her fads. Alas! scarcely had they been two days settled when the nurse reported, "There's illness upstairs, ma'am; but I can't find out what it is." The landlady refusing to say what was the matter, Mrs. R. grew suspicious, and then, to her intense horror, discovered it to be measles. Poor thing, she knew too much; a little knowledge is said to be dangerous, but as regards microbes, a small amount of knowledge is delightful and fascinating. It is only too much that is dangerous!

### Where to Go.

PARKES MUSEUM, MARGARET STREET.—Lectures on Domestic Hygiene, at three p.m., on March 2nd, 6th, 9th and 13th, by Dr. Schofield.

THE DUDLEY GALLERY.—The "private view" of the Dudley Gallery Art Society on Saturday was a "private" view in the sense that admission was by invitation only. The British public were but too well represented, and the show that day was rather a display of Persons than Pictures. But though difficult of approach, most of the water-colours, when seen, rewarded one for the effort it cost to do so. Of the President's work, No. 1, "Beech Trees, Forglen," being the most attractive, the general effect of chequered light and shade in the beech wood is especially good. In his "Quiet Day Before the Twelfth," there is a want of breadth, and more particularly a want of unity, which is to be accounted for, perhaps, when we bear in mind that this is the work of two artists, Mr. Walter Severn and Mr. Edward Neal. One is struck by the quantity and the quality of the work of the lady exhibitors, conspicuous among whom being Miss Helen Carlisle, Miss Margaret Bernard, Miss Nora Davidson, and Miss Evangeline Jex Blake. These lady artists have a decided strength and individuality about their work which promises to place them in a high rank amongst water-colourists of their day. Mr. Herbert Finn and Mr. David Green may be grouped with the above as artists who do not overload their work with detail, but delight us with their strong, fresh colouring. But the school which elaborates detail and subjects its work to an exhaustive "finish" is also not absent from the walls of the Dudley Gallery this year. Taken as a whole the thirtieth exhibition of this society's paintings is an especially good one, and thoroughly maintains its standard of general excellence.

### Everybody's Opinion.

[Correspondence on all subjects is invited, but we cannot in any way be responsible for the opinions expressed by our correspondents. No communications can be entertained if the name and address of the correspondent is not given, or unless one side of the paper only be written on.]

### A NURSES' CO-OPERATION.

A "Male Nurse" writes: I trust you will pardon my asking you to bring before your readers a few remarks on this subject on behalf of my fellow-workers. The report lately published of the annual meeting of the Nurses' Co-operation, 8, New Cavendish Street, is most satisfactory, and is no doubt greatly due to the kindly interest taken in it by those who first promoted the association; but, being only for female nurses, I would venture to ask whether this good service could not be extended to male nurses, who, though equally deserving, are mainly dependent upon agents, who pocket from 25 to 40 per cent. of the earnings. There are well-trained, efficient, and well-qualified men who would be willing to aid in the promotion of such an association, and as there are many cases in which male nurses are, or should be, preferred to female nurses, I feel sure that such a society would become not only profitable, but also a public boon.

### NURSING OVARIAN PATIENTS.

"Another Senior Nurse" writes: I also have had much experience in the nursing of ovariotomy and other abdominal cases, and think Mr. Dick's ideas are excellent. I have seen many cases in which the sheet did not get damp, and in that case it is usually the best means of lifting the patient to the bed; it would, of course, be taken out at once, the bed being already arranged with drawsheet and mackintosh. Again, where is a hand to be found to put on either side of the wound during vomiting if a woman is nursing the case alone, as I have always done? And if there is a glass tube, and neither dressing nor binder are to be touched, how is this to be attended to? I quite agree with Mr. Dick in saying that such patients are best without morphia, though there is more trouble for the nurse, as constant attention is required after recovery from the chloroform, but it is better in the end. As to the catheter, there are few cases where it is necessary. I have always passed the slipper during the first night, eight to ten hours after operation, sometimes earlier, even with a glass tube in the wound, and without bad results.

### MIDWIVES' REGISTRATION.

Dr. Hugh Woods writes: Dr. Alderson says that I wrote a letter on the above subject evidently under annoyance or irritability, for I had said, "There is no need for a Midwives' Bill, and there shall be none." This quotation is entirely derived from Dr. Alderson's imagination, or that of his modest medical friend who requests him to reply to me. I myself strongly supported Dr. Alderson's candidature for a seat on the General Medical Council, and quite approve of the views he then expressed on the midwives question. But Dr. Alderson then said what he himself thought, and he was not then speaking at the request of a prompting supporter of the Midwives' Bill. I give Dr. Alderson credit for genuine earnestness in his efforts to advance the interests of the general practitioner; but I think he will be wiser to listen to the counsel of his tried friends in the Medical Practitioners' Association rather than to that of men who try to persuade him that the public will never know in which column of a register, which will not pay the expenses of publication, any individual licensed to practise midwifery without the supervision of a medical man, may appear. I am sorry that Dr. Alderson has joined a society which can only exist by granting dignities free of charge.

### For Reading to the Sick.

**Motto.**

Sullenness.

Our faults are at the bottom of our pains.—*Young*.

**Verses.**

Of long and weary days,
Full of rebellious askings, for what end,
And by what power, without our own consent?
* * * * * *
We were placed here, to suffer and to sin,
To be in misery and know not why.
. . . Our own life seemed then
But as an arrow flying in the dark,
Without an aim; a most unwelcome gift,
Which we might not put by. —*R. C. Trench*.

From the ingrained fashion
Of this earthly nature
That mars thy creature;
From grief—that is but passion;
From mirth—that is but feigning;
From tears—that bring no healing;
From wild and weak complaining—
Thine old strength revealing—
Save, oh, save!
—*Matt. Arnold*.

If I have moved among my race,
And shown no glorious morning face;
If beams from happy human eyes
Have moved me not: if morning skies,
Books, and my food and summer rain
Knocked on my sullen heart in vain;
Lord, Thy most pointed pleasure take,
And stab my spirit broad awake;
Or, Lord, if too obdurate I,
Choose Thou before that spirit die,
A piercing pain, a killing sin,
And to my dead heart run them in.
—*R. L. Stevenson*.

**Reading.**

Different as winter from summer, as night from day, ay, even as death from life, looms the dreary, joyless, thankless, fruitless gloom of sullenness, the sour sorrow of the world, that wanton, wilful self-distressing that numbs all love and zeal for good; that sickly, morbid weariness in which the soul abhors all manner of meat, and is even hard at death's door—the sin that is opposed to the joy of love. Heaviness, gloom, coldness, sullenness, distaste and sloth in work and prayer, joylessness, and thanklessness—do we not know something of the threatenings at least of a mood of this sort? The mood of days on which it seems as though we cannot genuinely laugh, as though we cannot get rid of a dull or acrid tone in our voices; when it seems impossible frankly to "rejoice with them that do rejoice" . . . days when, as one has said, "everything that everybody does seems inopportune and out of good taste; days when there is nothing that we like to do—when, without anything to complain of, nothing stirs so readily in us as complaint. Oh! if we know anything at all of such a mood as this, let us be careful how we think of it, how we deal with it; for it may not be far from that "sorrow of the world" which, in those who willingly indulge and welcome and invite its presence, worketh death.—*Dr Paget, Dean of Christ Church.*

It occurs to one at once that this misery lies on the border line between the physical and the spiritual life; that if there is something to be said of it as a sin there is also something to be said of it as an ailment. . . . It is a truth which should make us endlessly charitable, endlessly forbearing and considerate towards others; but surely it is a truth that we had better be shy of using for ourselves. It will do us no harm to over-estimate the degree in which our own gloom and sullenness are voluntary. It will do us very great harm to get into the way of exaggerating whatever there may be in them that is physical and involuntary. . . . Surely it has been the secret of some of the highest, noblest lives that have helped the world that men have refused to make allowances for themselves. . . refused to take the easy tasks which their hindrances might seem to justify. . . Men "who going through the vale of misery use it for a well and the pools are filled with water." And "they shall go from strength to strength"—in all things more than conquerors through Him who loveth them.—*Dr. Paget.*

### The Book World for Women and Nurses.

[We invite Correspondence, Criticism, Enquiries, and Notes on Books likely to interest Women and Nurses. Address, Editor, THE HOSPITAL (Nurses' Book World), 428, Strand, W.C.]

LETTERS OF TRAVEL. By PHILLIPS BROOKS, late Bishop of Massachusetts. (London: MacMillan and Co., 1893. Price 8s. 6d. net.)

Bishop Phillips Brooks' travels were by no means remarkable. For an American of means and culture to visit Europe, Palestine, India, and Japan in these days implies no more than the possession of means and culture. If the Bishop had been a less popular man, with the highest kind of popularity, that of a kind and earnest nature, these letters would never have been published; and if so, no one would have been much the poorer. Yet, having them in our hand, we read them with pleasure, noting in them that quality of appreciation of the good in things English, combined with a patriotic loyalty to America, which is so conspicuous in such a man as Lowell, and so notably absent from the average travelling Yankee. Bishop Brooks came over with introductions to the best English society, and his comments and notes, though uncritical, are often interesting. Thus it is a pleasant addition to Tennysoniana to learn that the poet himself regarded the simile in "Locksley Hall"—

Love took up the lamp of life, and turned it in his glowing hands,

—as the finest he ever conceived. In face of Wendell Holmes's dictum that man is a racing as well as a praying animal, we may record that the Bishop went to the Derby. "The Prince of Wales was there and so was I," says he. His comments on Cambridge—"Our Alma Mater's Mater," as he calls it in writing to his brother, is not without value: "The students seem to me very like, indeed, to Harvard boys—the same average of age, the same general bearing, the same sort of talk. If anything especially gives them an advantage over us it seems to be in the University system, the grouping of colleges so as to create a friendly corporate as well as personal rivalry, and the presence among them of older and maturer scholars, residing on fellowships, &c., who raise the scholarly standards of the place higher than they could be set by mere undergraduate attainment. Both of these advantages, I think, are capable of being engrafted on our system, and if they ever are I see no reason why, in time, our greater freedom from old prescriptions and restraints should not make our University a better place than this." This book deserved publication, not as a record of travel, but as a record of a true, generous, and sympathetic man.

OUR DAILY FARE. (Ward, Lock, and Bowden, London.)

THE publishers of "Our Daily Fare" have provided a very useful and much-needed little book for household use. It contains, as is set forth on the cover, a practical series of bills of fare for meals throughout the year, costing from four shillings to seven shillings per head per week for households consisting of varying numbers of persons. Such a work has been wanting long, but the task is a difficult one; and, indeed, useful as the present little volume will be found both to the home housekeepers of long standing and to inexperienced ones, it is not free from some defects. The authoress, who by the way does, not appear in *propria personæ* throughout the pages, frankly admits the great difficulty she found in the task of catering for a family of six at four shillings a head. We quite allow that it is legitimate to omit any beverages other than tea and coffee for the adults from her weekly calculation for so small an expenditure, but we do not think she is wise in suggesting so small an amount as four quarts of milk per week for a family in which are numbered four children. This may be economy on paper, but will be hardly so in practice. Then for dwellers in towns the price quoted for vegetables will be found quite inadequate unless the home is situated in very cheap quarters. We fully expect the present little volume will pass speedily to a second edition, when, with a slight revision, these small defects in the plans put forth might be eliminated. We think, too, some definite hints where to go for commodities might be especially helpful for Londoners. The recipes at the end will be found excellently chosen, and the directions are simply clear. Taken as a whole the little volume is full of practical and useful information, and will form a welcome gift to the young housekeeper especially, and we hope before long to see a work from the same pen devoted to the wants of those who, with slightly larger incomes, require assistance in catering for lunch and late dinner as opposed to early dinner and supper.

---

### MAGAZINES OF THE MONTH.

COUNT TOLSTOI'S article on "The Preaching of Christ and the Practice of His Churches" last month has called forth abundant replies in the NEW REVIEW. The Bishop of Ripon, the Archdeacon of London, the Rev. J. Rickaby, and the Rev. J. Guinness Rogers have all taken pen in hand to dispute the Count's conclusions. Mr. Guinness Rogers is decidedly the most successful. The other three gentlemen would not appear to have studied any of Tolstoi's religious writings, with the exception of the article about which they have taken it upon themselves to write. Had they done so, they would surely have saved themselves the trouble of citing Moses and St. Paul to a man who declares that Christ came to annul the Mosaic Law and that St. Paul failed to perceive the true significance of his Master's teaching. Mr. Rogers, while deprecating Tolstoi's inconsistencies and fanaticism from the point of view of common sense, frankly admits that the attitude of the Church towards national war has been and is utterly unjustified by her teaching. In the same review, "Nauticus" urges the adoption of a scheme for the better education and training of our naval officers, whom he compares somewhat unfavourably with those of foreign navies, ascribing their deficiencies, in a great measure, to the early age at which they enter the service.

THE NINETEENTH CENTURY contains an article which will propably excite scorn and disgust in the minds of the advocates of women's suffrage. According to Mr. Bakewell, the women of New Zealand have no political opinions properly speaking. The only subjects they interest themselves in are the Prohibition and Education questions, on both of which they are rabidly prejudiced. Neither had they, in general, any desire for the suffrage. Mr. Bakewell is evidently an opponent of the party which has come into power on the votes of the women. Let us hope that the picture he has drawn is a little coloured by party prejudice.

SCRIBNER'S MAGAZINE has an article on Burne-Jones by Cosmo Monkhouse, profusely illustrated by reproductions of several of the painter's sketches and best-known works. "The Schoolmaster," by James Baldwin, is a record of the American country schoolmaster of bygone days. His life does not seem to have been always an easy one, and it is not surprising, considering the quality of his pupils, to hear that he often maintained discipline by the aid of an assortment of switches.

## Figures and Tables

**Figure f1:**